# *Sida cordifolia* is efficacious in models of Huntington’s disease by reducing ER stress

**DOI:** 10.3389/fmolb.2025.1567932

**Published:** 2025-03-19

**Authors:** Prasanna K. Simha, Chandramouli Mukherjee, Vikas Kumar Gupta, Karishma Bhatia, Padmanabhi Nagar, Azeem Nazeer ZA, Ashwini Godbole, Bhavani Shankar Sahu, Sanjeev K. Upadhyay

**Affiliations:** ^1^ Centre for Ayurveda Biology and Holistic Nutrition, University of Trans-Disciplinary Health Sciences and Technology (TDU), Bengaluru, India; ^2^ National Brain Research Centre, Gurugram, Haryana, India; ^3^ Department of Biochemistry, Faculty of Science, The Maharaja Sayajirao University of Baroda, Vadodara, Gujarat, India; ^4^ Department of Botany, Faculty of Science, The Maharaja Sayajirao University of Baroda, Vadodara, Gujarat, India; ^5^ Kerala Ayurveda Clinic, Bengaluru, India

**Keywords:** *Sida cordifolia*, neurodegeneration, Huntington’s disease, ER stress, ayurveda biology

## Abstract

Neurodegenerative disorders (NDs) are a major class of diseases where modern science has not succeeded in providing solutions to the desired levels. ER stress pathway is implicated in pathophysiology of several neurodegenerative disorders, especially those classified as proteinopathies. Several traditional medicines are used to treat neurodegeneration and *Sida cordifolia* (SC) is one of the common ingredients in formulations used for treating NDs and neuropathic pain. However, the mode of action is not clear. We studied the effectiveness of SC in Huntington’s Disease (HD) model using *Caenorhabditis elegans* and mammalian cells. We used a transgenic *C. elegans* that expresses mutant huntingtin protein tagged with Yellow Fluorescent Protein (YFP) in their body wall muscle. In *C. elegans*, SC not only improved motility but also substantially increased the life span. Cell-based studies using inducible mutant Huntingtin protein (mHTT) with a long polyQ tail tagged with EGFP showed that SC profoundly modulates ER stress, reducing the stress caused by mHTT protein. The study showed that the mode of action of SC, at least partially, is through modulation of ER stress pathway, thereby normalizing the changes brought about by overexpression of mHTT.

## 1 Introduction

Huntington’s Disease (HD) is an autosomal dominant neurodegenerative disorder caused by the expansion of CAG trinucleotide repeats in the huntingtin (*Htt*) gene on chromosome 4. The disease is estimated to affect about 1 in 7,500 individuals in the population (Caron et al., 2018), being more prevalent in the Western population than in Asians (McColgan and Tabrizi, 2018). The mutant genes encode huntingtin protein with long tandem repeats of poly-glutamines (polyQ), typically more than 35 in a stretch. Longer CAG repeats cause earlier onset of disease and increased severity (McColgan and Tabrizi, 2018). The mutant protein misfolds and aggregates which is a cause for neurodegeneration (McColgan and Tabrizi, 2018). Cognitive, motor and psychological disturbances are attributed to neuronal degeneration in the striatum, globus pallidus, subthalamic nucleus, thalamus, substantia nigra and hypothalamus of the brain (Caron et al., 2018).

Current treatments for HD are symptomatic and do not rectify the underlying causal cellular and molecular derangements. Symptomatic therapies include anti-dopaminergic agents and occasionally anti-depressants. Prolonged usage of these treatments in HD patients is associated with adverse effects (Ferguson et al., 2022) including diarrhea, nausea, vomiting, depression, Parkinsonism and suicidal tendencies (Paleacu, 2007; Yero and Rey, 2008). In animal models, azo-dye congo-red (Sánchez et al., 2003), disaccharide trehalose (Tanaka et al., 2004), and polyQ-binding peptide-1 (Nagai et al., 2003) have been reported to decrease the aggregation of mutant huntingtin protein, but none of these can cross the blood-brain barrier, impeding their development as therapeutics. Taken together, there remains an unmet need for new therapies to rectify the molecular derangements in HD, namely the aggregation of mutant huntingtin proteins and the toxic effects they cause in neurons.

Many cellular signal transduction pathways are implicated in HD. One cellular mechanism implicated in protein misfolding disorders involve the Endoplasmic Reticulum Unfolded Protein Response (ER-UPR). Studies have shown that chronic activation of the ER-UPR is a common feature of these diseases. In mammals, three main pathways, initiated by three different type-1 transmembrane proteins, namely: inositol – requiring protein – 1α (IRE -1α), protein kinase RNA-like ER kinase (PERK) and activating transcription factor-6 (ATF6), govern the ER-UPR. Manipulating these pathways using small molecules or genetic tools have shown to be effective in models of neurodegeneration (Hughes and Mallucci, 2019). In the case of HD, targeting XBP-1 is shown to be beneficial in animal models, and is thus being explored as a potential drug target (Vidal et al., 2012).

Ayurveda, an Indian traditional system of medicine has the pharmacopoeia of approximately 70,000 formulations. *Sida cordifolia* (Flannel Weed, *Bala*) (SC), a perennially growing shrub of the mallow family *Malvacea* native to India, is one of the most commonly used herbs in Ayurveda to treat diseases of the nervous system.

Ayurvedic formulations containing SC have been effectively used to treat pain, neurological diseases associated with tremors or movement disorders (Sati and Deole, 2020), Parkinson’s Disease (Anjaneyulu et al., 2020) and arthritis (Das et al., 2021). Many of these ailments are degenerative in nature. Interestingly, in a case study with one patient of early onset HD, whole-system Ayurvedic treatment lead to reduction in involuntary movements, improved balance and gait (Malavika and Savitha, 2022). SC formed an important component of the treatment. This case report and observations from the practice of Ayurveda inspired us to determine if SC could reduce aggregation of mutant huntingtin protein in models of HD. It is interesting to note that in all the ailments, for which SC is prescribed in Ayurveda, ER-UPR is a key cellular pathway in their pathogenesis.

*Acorus calamus* (Sweet Flag, Muskrat root, *Vacha*) (AC), a plant from the family *Acoraceae*, is another neuroactive herb commonly used in Ayurveda. Unlike SC, AC is used for enhancing intelligence and treating epilepsy, speech disorders, which are non-degenerative in nature (Chunekar, 2004).

Here, we assessed the effect of methanolic extract of SC on cellular and molecular hallmarks in HD models. We chose to use AC, a differentially used herb, as a control to confirm the specificity of the SC effects. We used an animal model and a cell line model of HD. In *Caenorhabditis elegans* strains expressing a mutant huntingtin protein containing 40 tandem glutamines, the methanolic extract of SC, but not of AC, reduced aggregates of the polyQ-containing mutant huntingtin protein and thereby improved motility and prolonged life span of the nematode. In a transgenic mouse neuroblastoma cell line over-expressing a mutant huntingtin protein with 150 tandem glutamines, SC reduced aggregates of the polyQ-containing mutant protein by suppressing the Endoplasmic Reticulum Unfolded Protein Response (ER-UPR) triggered by aggregation. The results indicate that SC can be a source of novel therapeutics to inhibit aggregation of misfolded huntingtin proteins and the resulting neurodegenerative sequelae.

## 2 Materials and methods

### 2.1 Preparation of methanolic extract of *S. cordifolia* and *A. calamus*

Roots of SC (Specimen no. SANJ20/1) were obtained from herb collectors and rhizomes of AC (Specimen no. SANJ20/2) were obtained from Botanical Garden, MSU Baroda. The plant material was authenticated by Baro herbarium, Department of Botany, MSU Baroda.

The roots or rhizomes were thoroughly washed and dried, followed by powdering them coarsely. The plant root/rhizome powder and methanol (1:10 (w/v) root powder: methanol) was used for extraction using the soxhlet apparatus for 7–8 h. The extract was concentrated, and solvent was removed using a rotary evaporator and stored at −20°C for further use. TLC was run to check the quality of the extract. MTT assay was also performed to assess the toxicity (Supplementary Figure 5).

### 2.2 Culture and maintenance of *C. elegans*

The *C. elegans* strains N2 (wild type) and AM141 (rmls133 (unc54p::Q40::YFP); expressing Q40::YFP in body wall muscles of the nematode were procured from the *Caenorhabditis* Genetics Centre (CGC), University of Minnesota, United States. The worms were grown at 20°C on Nematode Growth Medium (NGM) seeded with *Escherichia coli* OP50 using standard protocols. All worm experiments were conducted in accordance with the internationally accepted principles and approved by institutional committee (IBSC/TDU/05/12/22).

### 2.3 Culture of N2a neuroblastoma cells over-expressing a mutant huntingtin protein with expanded polyQ, and treatment with extracts of *S. cordifolia*

Transgenic mouse N2a cells expressing an ecdysone-inducible truncated N-terminal huntingtin gene with 150 CAG repeats attached to EGFP (HD 150Q) were a kind gift from Professor Nihar Jana’s group, National Brain Research Center, Manesar, India. They were used to evaluate the effects of the *S. cordifolia* extract on huntingtin aggregates. The cells between passage numbers 2 to 10 were used for all experiments. They were cultured in 10% (v/v) FBS in DMEM, with 0.4 mg/mL Zeocin and 0.4 mg/mL G418 to its final concentration. For immunoblot experiments, 2 × 10^5^ cells were plated in 6-well plates in replicates. Induction (with 1 µM Ponasterone A) and treatment (1, 5, and 10 μg/mL SC methanolic extract) were started simultaneously 24 h after plating. Media was replaced with fresh media every 24 h for 2 days, and after 48 h of initial treatment, the cells were harvested and lysed for immunoblot or PCR (Figure 4A).

### 2.4 PolyQ aggregates – imaging

#### 2.4.1 In transgenic *C. elegans*

Age-synchronized AM141 worms were grown from embryo stage to Day 5 adult stage on freshly prepared NGM-OP50 plates (60 mm diameter) spotted with various concentrations (0.1 μg/mL, 1 μg/mL and 10 μg/mL) of methanolic extracts of SC or AC or vehicle control (DMSO). On Day 1, Day 3 and Day 5 of adulthood few of the worms were washed in M9 buffer and collected in sterile 1.5 mL microfuge tubes. 20 µL of 5 mM sodium azide solution was added to the worm suspension to paralyze them. The worms were then mounted on glass slides containing a 2% agar pad and covered using a cover slip (Pigazzini et al., 2020). The immobilized worms (up to 10 worms per group) were observed under a fluorescent microscope (Olympus BX41) equipped with a camera (Olympus DP72). The images were captured under a blue filter (excitation wavelength 455 nm–495 nm) using the Image-Pro Express software. The aggregates were defined as discrete structures with clear boundaries for counting puncta and quantified by measuring the average fluorescence intensity in the head region of the worm (up to the second pharyngeal bulb). The remaining worms were transferred to respective fresh drug containing/control plates every day to avoid starvation and mixing of the consecutive generations.

#### 2.4.2 In cells over-expressing mutant huntingtin protein

2 × 10^4^ cells were seeded in each well of 4 chamber slides, and after completion of treatment, cells were washed gently with PBS twice and then fixed using 4% PFA for 20 min. Thereafter, cells were mounted using DAPI containing mounting media (Fluorshield with DAPI SIGMA F6057-20 mL) using 24 mm*40 mm cover glass (blue-star) and imaged on the next day in 40x oil Zeiss-Apotome microscope.

### 2.5 Lifespan assay

Age-synchronized AM141 worms were grown on freshly prepared NGM-OP50 plates (60 mm diameter) spotted with various concentrations of preparation of *S. cordifolia* or *A. calamus* or vehicle control (DMSO), up to late L4 stage. The worms were then transferred to fresh drug-containing/control NGM-OP50 plates every day until all worms in the group were dead. Worms were considered dead if they did not move after being lightly prodded using a platinum wire worm pick (Cooper et al., 2015). Data was analyzed using OASIS 2 software to obtain Kaplan-Meyer survival curves and mean/median lifespan values (Han et al., 2016).

### 2.6 Thrashing assay

Age-synchronized N2 or AM141 worms were grown on control plates or on plate spotted with various concentrations of methanolic extracts of *S. cordifolia* or *A. calamus*, up to Day 5 adulthood. The worms were transferred to fresh drug containing/control NGM/OP50 plates every day using a worm pick. They were collected in M9 buffer and suspended in a 15 µL droplet of the buffer on a glass slide. Thrashing of the worms (up to 10 worms per group) was video recorded using a Levenhuk lite microscope camera, on days 1, 3 and 5 of adulthood. The thrashing was analyzed by counting the body bends manually (Currey and Liachko, 2021).

### 2.7 Worm development assay

Age-synchronized wild type (N2) worms were grown on fresh NGM/OP50 plates containing various concentrations (0.1 μg/mL, 1 μg/mL, 10 μg/mL) of methanolic extracts of *S. cordifolia* or *A. calamus* or vehicle control (DMSO) at 20°C from the embryo stage to Day 1 of adulthood. The larval stages 1, 2, 3, 4 and day 1 adult worms were imaged under Olympus IX71 microscope using the Olympus DP72 camera, at 26.5, 36, 45, 56, and 65 h respectively, from the embryo stage. Lengths of at least 10 worms per group were measured at each stage, using ImageJ software (Lee and Kang, 2017).

### 2.8 Worm fecundity assay

Age-synchronized wild type (N2) worms were grown on fresh NGM/OP50 plates containing various concentrations (0.1 μg/mL, 1 μg/mL, 10 μg/mL) of methanolic extracts of *S. cordifolia* or *A. calamus* or vehicle control (DMSO) at 20°C from the embryo stage to late L4 stage. 5 late L4 worms per group were then transferred to 5 fresh NGM/OP50 plates containing respective drugs or vehicle control, such that there was one worm per plate, and allowed to grow at 20°C, for the rest of the worm’s reproductive cycle (days 1–5 of adulthood). The worms were transferred to fresh plates every day and the number of eggs laid per worm were counted each day under the microscope and the total number of eggs laid per worm throughout its reproductive cycle was counted (Ha et al., 2022).

### 2.9 Immunoblotting

Following completion of treatment, cells were lysed with RIPA lysis buffer (50 mM Tris, pH 7.4, 150 mM NaCl, 1 mM EDTA, 0.1% SDS, 1% Triton X-100, 1% sodium deoxycholate with, 1 mM phenylmethylsulphonyl fluoride, 2 mM Na_2_VO_5_, 1 mM NaF, 20 mM Na_4_P_2_O_7_ as a phosphatase inhibitor cocktail) at 4°C. Lysates were collected, centrifuged at 14000 ×g for 15 min and the supernatant was collected. Total protein was estimated using the BCA (Bicinchoninic Acid) method. 20 µg protein was loaded on SDS-PAGE and transferred to a nitrocellulose membrane. Membrane was blocked with 5% skimmed milk or 3% BSA (for phosphoprotein) for 2 h at room temperature. Membranes were then probed overnight with primary antibodies of anti-GFP (rabbit, 1:5,000), anti-GAPDH (Mouse, 1:8,000), anti-pEIF2α (rabbit, 1:2,500), anti-EIF2α (rabbit, 1:5,000), anti-IRE1α (Rabit, 1:5,000), Anti pIRE1α (rabbit, 1:2000), and washed with Tris-buffered saline, 0.1% Tween (TBST) and probed with HSP-tagged secondary antibody targeted against the host species of the primary (1:10,000). Blots were developed using Luminol and chemiluminescence images were captured using the Uvitec Cambridge gel doc instrument.

### 2.10 Quantitative polymerase chain reaction (qPCR)

Total RNA was extracted using TAKARA RNAiso, following the manufacturer’s protocol. cDNA was prepared using BioRad iScript 5x RT supermix in a single-step reaction. qPCR was performed using iTaq SYBR green (BioRad) following the manufacturer’s protocol in CFX96 (BioRad). In another set of reactions, the synthesized cDNA was used to check the expression of the GFP transcript by semiquantitative PCR using GTPCR master mix (TAKARA BIO) for the GFP-specific primer, normalized with GAPDH. Primers are listed in Supplementary Table 1.

### 2.11 Statistical analysis

Statistical comparisons between two groups were performed with students’ unpaired t-tests when data were normally distributed with similar variances; otherwise, the Mann-Whitney test was performed to draw the significance. A p-value of 0.05 or less was considered statistically significant. Statistical analysis was performed using GraphPad Prism 8.0. The data are presented as means, and error bars show the standard error of the mean (SEM).

## 3 Results

### 3.1 Methanolic extract of SC inhibits aggregation of polyQ-expanded mutant huntingtin protein in the *C. elegans* model of HD

**FIGURE 1:**
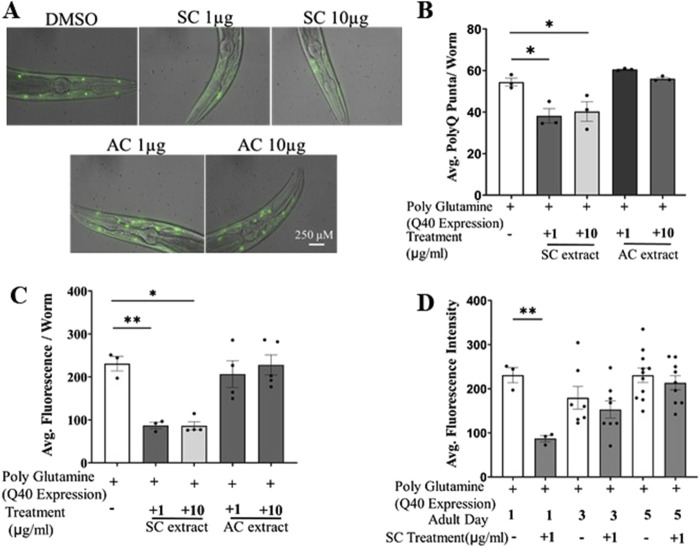
SC reduces polyQ aggregates in transgenic HD model of *C. elegans* (AM141). **(A)** Representative fluorescent microscopy images (day 1 adult worms) of poly Q aggregation in AM141 worms treated with SC or AC extracts **(B)** quantification of number of poly-Q puncta per worm (day1) (n = 3; for all the conditions) **(C)** average fluorescence intensity of poly-Q per worm **(D)** average fluorescence intensity of poly-Q in Day 1, Day3 and Day5 adult worms. (In all the graphs, each dot represents the mean of one experiment and a minimum of 3 means were considered for each graph. For each experiment 10 worms were used per group. Data represented as mean ± SEM, *p < 0.05, **p < 0.01).

The toxic aggregation of misfolded mutant huntingtin proteins with expanded tandem repeats of polyQ in the central nervous system is the pathological hallmark of HD. The transgenic *C. elegans* strain expressing polyQ proteins is an excellent, widely established model studying cellular effects of aggregated proteins (Van Pelt and Truttmann, 2020). We investigated the possible therapeutic effect of SC and AC, in the AM141 strain of *C. elegans* which expresses a mutant huntingtin protein with 40 tandem glutamines (Q40::YFP) in body wall muscles (Morley et al., 2002). These worms accumulate the polyQ protein-aggregates as they age, which are seen as fluorescent punctae in the muscles (Figure 1A). We cultured these worms on fresh NGM/OP50 plates spotted with methanolic extracts of SC or AC. Worms treated with the SC extract, from embryo stage to day 1 adult stage (72 h at 20°C), showed about 20%–30% reduction in the number of YFP puncta (a measure of aggregated mutant huntingtin protein) as compared to vehicle/solvent (DMSO)-treated worms (Figures 1A, B; Supplementary Figure 1A). The average fluorescence levels of the puncta were also lower upon SC treatment (Figure 1C). This effect was specific to the SC extract, as the methanolic extract of AC did not reduce mutant huntingtin protein aggregation. We next performed imaging-based quantification of polyQ aggregates in worms at days 1, 3 and 5 of adulthood. Worms treated with 1 μg/mL SC significantly reduced fluorescence intensity of polyQ-YFP on day 1, and this effect gradually dissipated by day 5 (Figure 1D). Taken together, these results show that the SC extract (1 μg/mL) reduces aggregation of the mutant polyQ huntingtin protein. The treatment did not alter the development of the worms or their egg laying capacity (Supplementary Figures 1B, C).

### 3.2 SC treatment improves motility in the *C. elegans* model of HD

**FIGURE 2:**
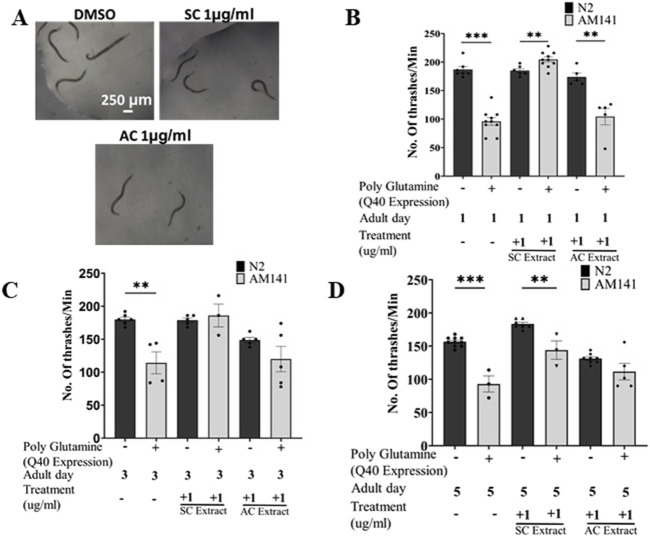
SC improves motility in HD model of *C. elegans*. **(A)** Representative images of thrashing of Day 1 adult AM141 worms Thrashing assay of N2 (wild type) and AM141 (HD model) worms treated with SC or AC extracts. Quantification of thrashing of **(B)** Day 1, **(C)** Day 3, and **(D)** Day 5 adult AM141 worms. (In all the graphs, each dot represents the mean of one experiment and a minimum of 3 means were considered for each graph. For each experiment 10 worms were used per group. Data represented as mean ± SEM, **p < 0.01 *p < 0.05).

*Caenorhabditis elegans* swim by producing alternating C-shape and straight conformations called “thrashing” (Gjorgjieva et al., 2014). Aggregation of polyQ mutant huntingtin proteins in the body wall muscle leads to defects in thrashing. We performed the thrashing assay to investigate whether SC treatment improved motility of HD worms through its reduction of aggregated mutant huntingtin proteins. Worms grown on fresh NGM/OP50 plates containing different concentrations of SC or AC extracts were video graphed and their motility checked with the thrashing assay on days 1, 3 & 5 of adulthood. Treatment with SC (1 μg/mL), but not AC, restored motility of mutant worms to that of wild-type worms on day 1 (Figures 2A, B), the effect decreasing by day 5 (Figures 2C, D; Supplementary Figure 2).

### 3.3 SC treatment extends life span of HD model of *C. elegans*

**FIGURE 3:**
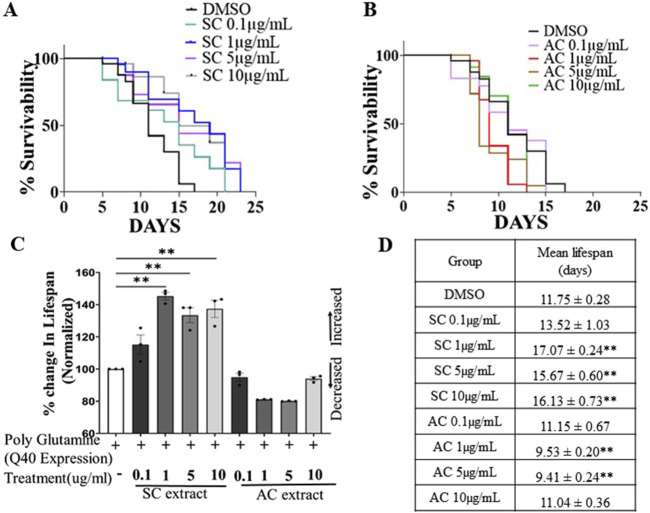
SC improves lifespan of HD model of *C. elegans*. Survival curves of AM141 (HD model) worms **(A)** treated with SC extract **(B)** treated with AC extract. **(C)** Quantification of percentage change in lifespan of AM141 worms treated with SC or AC extracts n = 3 for all the condition). **(D)** Lifespan assay data of AM141 worms treated with SC or AC extract. n = 25 per group. Data are mean ± SD. *p < 0.05 **p < 0.01 and n-Individual Biological replicates).

Earlier studies showed that lifespan extension in neurodegenerative models of *C. elegans* is neuroprotective (Cooper et al., 2015). We investigated whether SC would extend the lifespan of *C. elegans*. Worms were treated with different concentrations of SC or AC extract on NGM OP50 plates from the embryo stage. SC extended the life span of AM141 worms expressing the 40Q mutant huntingtin protein, from 11.55 days in untreated worms to 17.07 days in SC-treated (1 μg/mL) worms (Figures 3A, D). In contrast, treatment with AC reduced life span of these worms (Figures 3B–D).

Although the fluorescence levels in worms are no different upon chronic (by day 5, Figure 1D) treatment with SC, the benefits of treatment last much longer as the worms are more motile (Figures 2C, D; Supplementary Figure 3) and live longer.

### 3.4 SC reduces aggregation of mutant polyQ-containing huntingtin proteins in a mammalian system by reducing ER stress

**FIGURE 4:**
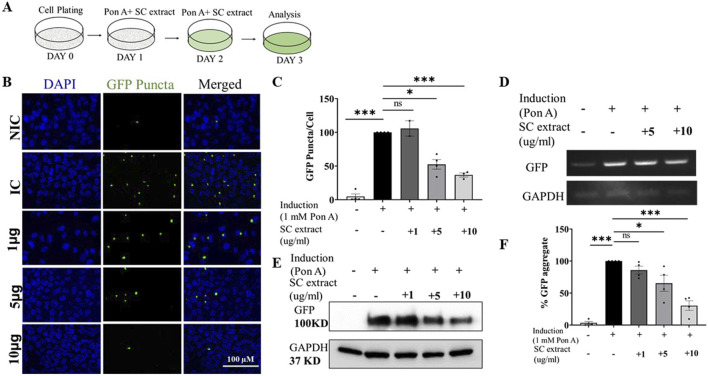
SC reduces protein aggregate in the poly-Q-EGFP expressing cells **(A)** Treatment protocol **(B)** Representative images of SC treated cells **(C)** Quantification of GFP-puncta per cell. (NIC, n = 3; IC, n = 4 SC 1 μg, n = 2; SC 5 μg, n = 4; SC 10 μg, n = 3) **(D)** Semi-quantitative PCR of GFP mRNA expression **(E)** Immunoblot of GFP protein **(F)** Quantification of immunoblots of GFP (n = 4 for all conditions). All data represented as mean ± SEM, *p < 0.05, **p < 0.01, ****p < 0.001 and ns is statistically non-significant and n = Individual biological replicate).

We tested if SC could suppress aggregation of mutant huntingtin proteins in a mammalian system. For these studies we used the mouse transgenic N2a neuroblastoma cell line over-expressing an ecdysone-inducible mutant Huntingtin protein with an expanded polyQ repeat (150Q) attached to EGFP (HD150Q-EGFP) (Wang et al., 1999). Upon treatment of these cells with 1 µM Ponasterone A, analog of Ecdysone (Figure 4A), the HD150Q-EGFP mutant protein aggregated, visualized as increased GFP puncta by fluorescence microscopy. SC treatment significantly reduced HD150Q-EGFP aggregates (Figures 4B, C) by decreasing mutant protein levels (Figures 4E, F) without altering mRNA levels (transcription) (Figure 4D). We did not notice any change in puncta sizes (Supplementary Figure 4A).

We next determined if SC-mediated reduction of mutant huntingtin protein aggregation was due to modulation of the Endoplasmic Reticulum Unfolded Protein Response (ER-UPR). In mammals, three main pathways initiated by three different type-1 transmembrane proteins: inositol – requiring protein – 1α (IRE -1α), protein kinase RNA-like ER kinase (PERK) and activating transcription factor-6 (ATF6), govern ER-UPR. XBP-1, a transcription factor critical in this process, is induced by ATF6, spliced by IRE-1, and modulating XBP-1 is beneficial in animal models of HD (Vidal et al., 2012).

**FIGURE 5:**
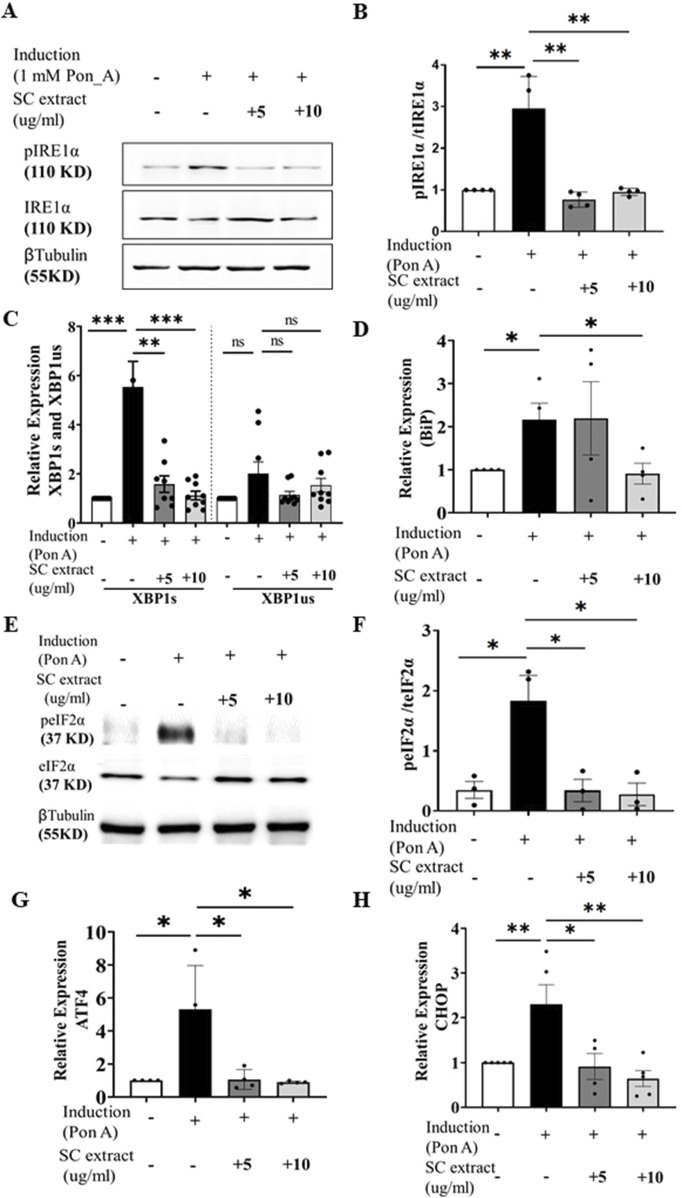
SC modulates ER stress pathway. **(A)** immunoblots of pIRE1α and IRE1α **(Β)** and its quantification (n = 4 for all conditions) **(C)** Relative expression of XBP1s and XBP1us (n = 8 for all groups except SC10µg where n = 9) **(D)** Relative expression of bip (n = 4, for all conditions) **(E)** Representative Immunoblot of pEif2a and total Eif2a, **(F)** its quantification n = 3 for all condition), **(G)** Relative expression of ATF4 (n = 4 for all conditions) and **(H)** CHOP. (n = 4 for all conditions) (All data represented as mean ± SEM, *p < 0.05. **p < 0.01, ****p < 0.001 and ns is statistically non-significant).

Ecdysone-induced aggregation of HD150Q-EGFP protein triggered ER-UPR as evidenced by increased phosphorylation of IRE-1α (Figures 5A, B), splicing of XBP-1 (Figure 5C) and activation of phospho-PERK-dependent phosphorylation of eIF2A (Figures 5E, F). Expression levels of BiP, an important chaperone in the ER stress response, was also reduced by SC treatment (Figure 5D). SC also suppressed other ER stress responses induced by HD150Q-EGFP protein aggregation including reduction of transcript levels of ATF4 and CHOP (Figures 5G, H). However, we did not find significant difference in GADD34 expression levels (Supplementary Figure 4B). CHOP, a protein linked to apoptosis (Oyadomari and Mori, 2004), is a key link between the ER stress pathway and apoptosis. SC-mediated reduction in CHOP raises the possibility that SC may inhibit apoptosis in stressed cells as a feature of neuroprotection.

## 4 Discussion

**FIGURE 6:**
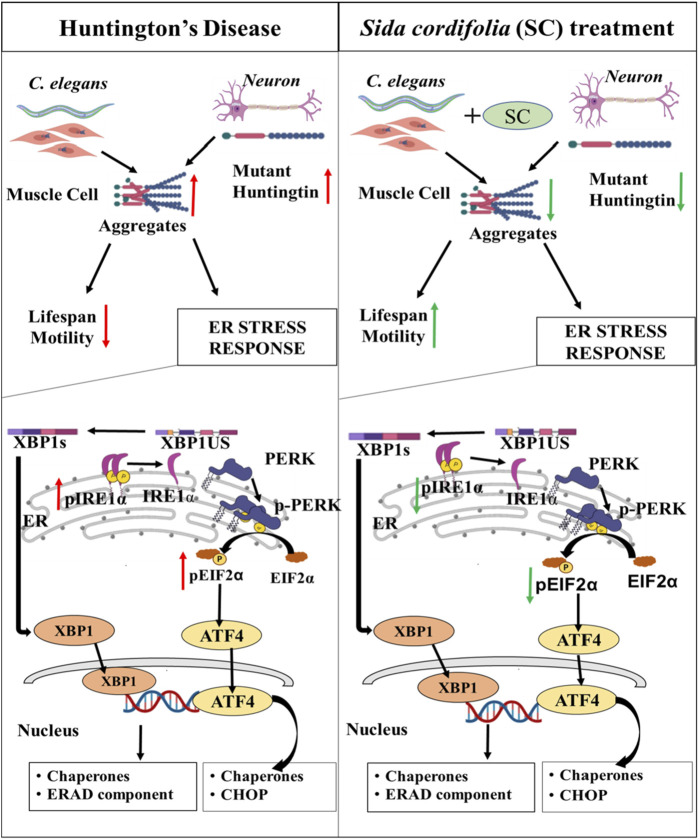
*Sida cordifolia* is efficacious in HD models by reducing ER stress. Reduces the aggregates in transgenic HD worm model expressing mutant protein (Q40::YFP) in their body wall muscle. This improves motility and enhances the lifespan of these worms. In neuroblastoma cells (N2a) expressing polyQ-EGFP SC treatment reduces the amount of mutant protein and thereby reducing the number of aggregates. This is accompanied by reduction in levels of key ER stress markers - Spliced XBP1s, pEIF2α and pIRE1α.

Here, we have explored the possibility of using Ayurvedic formulations for management and treatment of HD. Ayurveda uses SC containing formulation to treat neurodegenerative disorders, mainly targeting the weakness. We explored benefits of SC using an animal model of HD and suggest a mechanism based on the reduction of ER stress for the therapeutic effect. The extract’s mode of action, as indicated by our results is highlighted in a schematic in Figure 6. The extract likely contains several active ingredients that modulate multiple therapeutic targets. Such a multi-target therapeutic approach over single-target drugs is in line with current strategies being developed by the pharmaceutical industry. Future studies using fractionation of the extract coupled with cell and animal-based studies may help to identify active moieties within the extract and the molecular targets they modulate.

Huntington's, Alzheimer's, and Parkinson's disease are the most prevalent neurodegenerative disorders worldwide, for which many causative factors such as ageing, aggregation of certain proteins and specific genetic mutations are implicated. These factors lead to some common and distinct pathophysiological and clinical hallmarks. The research to develop treatment strategies often targets causative factors, like protein aggregation at cellular and molecular levels. Modern medicine’s current treatment strategies for neurodegenerative diseases are mainly oriented towards symptomatic care. Therefore, there is an unmet need to develop sustainable solutions for management of neurodegenerative diseases. One of the efforts in this direction is exploring traditional knowledge of healthcare and medicine.

Many herbs and formulations have been used effectively for centuries for treatment of neurodegenerative and neurological diseases, but the bio-medical evidence for the effectiveness and mode of action is largely lacking. Based on knowledge of traditional medicine and modern drug discovery approaches, one can explore possibilities of finding newer molecules from scientifically validated herbal formulations (Rahmati et al., 2018). Investigating and verifying the efficacy of these formulations could help us understand and establish them as potential therapeutics, especially for current unmet health needs.

Taking clues from the concept and practice of Ayurveda, we hypothesized the effectiveness of SC in the chronic age-related process like protein aggregation and neurodegeneration. On the other hand, AC is used for non-degenerative neurological conditions and was hypothesized to have limited benefits on HD models. The results from the experiments in the study corroborated well with the hypothesis. SC showed protective effects in the transgenic Huntington’s Disease model of *C. elegans*. Expression of polyQ disrupts the normal locomotor capacity of the worms, which was largely restored by SC treatment in HD worms (Figure 6). Furthermore, we show that SC increases the lifespan of the polyQ worms. Similar effects were not observed with treatment of AC. Surprisingly, worsening of movement in the worms treated with AC was observed on day 5 (Figure 2D). AC is described as very potent and excitatory, and the observed effects might be excitotoxicity. This will need further investigation.

The protective effect of SC was also visible in cellular models of HD (Figure 6). Using Neuro 2a cells expressing PolyQ-EGFP, we confirm that SC treatment reduces the polyQ protein amount and aggregation, as seen by immunoblotting and fluorescence microscopy respectively. We found that the SC affects at the post-transcription step in a fashion that reduces the total protein load of the PolyQ-EGFP, and consequently, reduces the aggregates. The reduction of aggregates could be either the reduction in translation or the result of the removal of aggregates by the cells’ stress-handling machinery. The reduction in translation looks less likely as the levels of p-eIF2α are reduced by SC treatment. Our study demonstrates an increase in the activity in IRE1α and eIF2α upon over-expressing the mutant HD protein. SC treatment reverses the pathway towards the control level as phospho-IRE1α and phospho-eIF2α protein levels revert to control levels. We observe these effects are translated to their respective downstream molecules, like XBP1s (downstream of IRE1α) and ATF4 and CHOP (downstream of eIF2α). The significant reduction in CHOP, a protein linked to DNA damage-induced apoptosis (Oyadomari and Mori, 2004) becomes very important as this is a key link between ER stress pathway and apoptosis. This observation is significant as it suggests that SC might reduce toxic effects by directly interfering with the apoptosis pathway in stressed cells for its neuroprotective effects. BiP, one of the primary players in the UPR and a key chaperone of ER, has been observed to be upregulated in stress conditions in several studies (Morris et al., 1997; Lei et al., 2017). Transcriptional regulation of genes to restore proteostasis in the ER is mediated by the XBP1 and ATF-6 branches of the UPR. Also, it is suggested that both BiP and XBP1 are target genes for ATF6. SC-mediated reduction in XBP-1 level agrees with the earlier finding where the loss of XBP1 was protective in the transgenic murine model of HD (Vidal et al., 2012). We guess these effects are because of enhanced clearance of the misfolded or aggregated protein, which needs further study. Also, we cannot distinguish between a mHtt specific effect or a general increased clearance of misfolded protein. Similarly, it remains to be seen whether the clearance is because of lysosomal or proteasomal degradation.

We noted a difference in effective doses in cell culture and *C. elegans*. We believe this might be due to different metabolizing enzymes or efflux pumps in worms and mammalian cell systems. The extract is more potent in the worms compared to N2a cells. Furthermore, molecular studies were done in the cell culture system partially because of possible signal dilution when we made protein extract or isolated mRNA from worms, as the body wall cells expressing transgenic protein form a small fraction of the entire worm.

Indian traditional medicine describes the role of SC in arthritis. A recent review enlists ER stress players as key targets for existing and potential drugs (Rahmati et al., 2018) in line with our findings. It is reassuring to see the specificity of the effect of herbs (SC and AC) in different types of biological conditions in agreement with the traditional knowledge. The methanol extract of SC likely contains several active ingredients that modulate multiple molecular targets. Future studies may help to identify active moieties within the extract and the molecular targets they modulate.

In summary, SC treatment reduces the aggregation of mutant polyQ-containing huntingtin proteins both in a *C. elegans* animal model and in a mouse neuroblastoma cell line. These effects are, at least in part, due to the suppression of various pathways in the ER stress response. Previously described experimental medications that reduced aggregation of mutant huntingtin protein in animal models (Nagai et al., 2003; Sánchez et al., 2003; Tanaka et al., 2004) had to be administered directly into the central nervous system because they were not able to penetrate the blood-brain-barrier. In contrast, the SC extract was effective in reducing aggregation of mutant huntingtin protein in *C. elegans* when applied into the growth medium, suggesting that it readily crosses the blood-brain-barrier. Further, oral administration of a SC-containing Ayurvedic formulation to a patient with juvenile-onset HD improved motor function, balance and gait without any adverse effects (Malavika and Savitha, 2022), again suggesting that SC-containing formulations can cross the blood-brain barrier giving hope for a safe therapeutic option for HD.

### 4.1 Preprint

A preprint has previously been published at bioRxiv (Simha et al., 2023).

## Data Availability

The original contributions presented in the study are included in the article/Supplementary Material, further inquiries can be directed to the corresponding authors.
