# Supplementary material for: Sida cordifolia is efficacious in models of Huntington’s disease by reducing ER stress
Source: Front Mol Biosci. 2025 Mar 19;12:1567932. doi: 10.3389/fmolb.2025.1567932 (PMC11961963; doi:10.3389/fmolb.2025.1567932)

Sida cordifolia is efficacious in models of Huntington's disease by reducing ER stress

Prasanna K Simha[1]*, Chandramouli Mukherjee[2]*, Vikas Kumar Gupta[3], Karishma Bhatia[3], Padmanabhi Nagar[4], Azim Nazeer ZA[5], Ashwini Godbole[1$], Bhavani Shankar Sahu[2$], Sanjeev K Upadhyay[1,3$]

S1

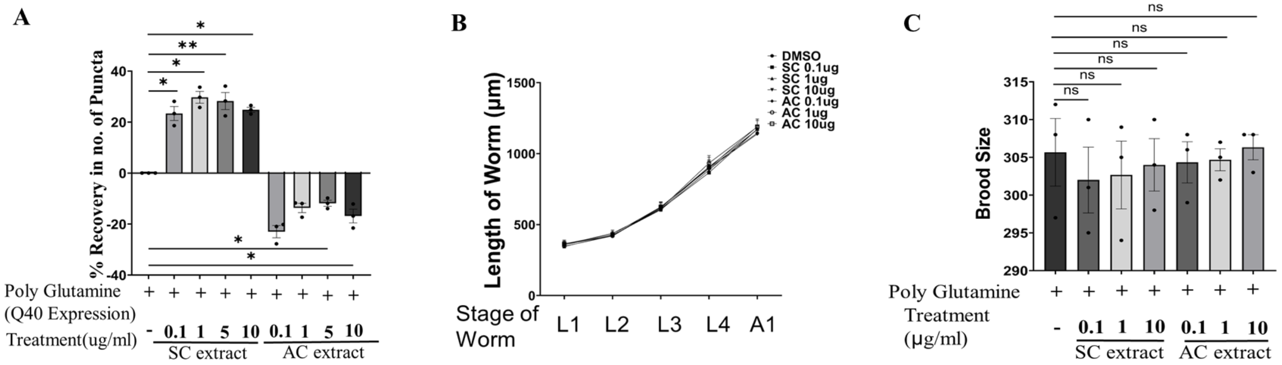

Supplementary Figure S1: SC reduces PolyQ in HD model of *C. elegans* without altering growth or egg laying ability. Percent recovery data in terms of number of puncta per worm on Day 1 (Each dot is a mean reading from 10 worms, n=3) (A), size of worms (B) (minimum 20 worms/group/stage), and brood size (C) upon treatment with SC or AC (Each dot is mean reading from 5 worms, n=3). *p<0.05, **p<0.01. Data are mean ± SEM and n = Individual biological replicates.

S2

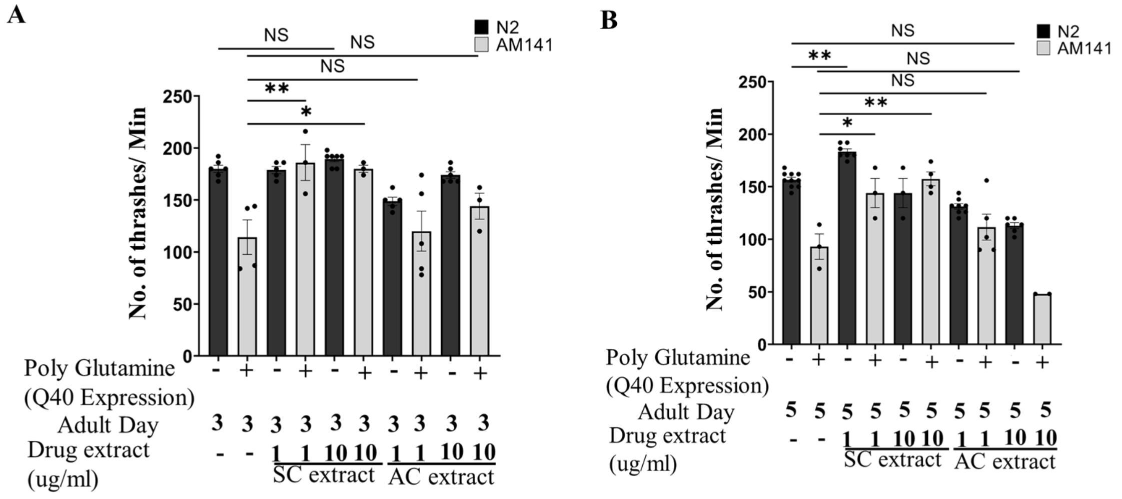

Supplementary Figure S2: SC improves motility in HD worms. Quantification of thrashing assay of day 3 (A) and day 5 (B) in adult AM141 and N2 worms treated with SC or AC extract. (In all the graphs, each dot represents the mean of one experiment and a minimum of 3 means were considered for each graph. For each experiment 10 worms were used per group. *P<0.05, **p<0.01. Data are mean ±SEM)

S3

Group	Median Lifespan (Days)	SD
DMSO	11.67	0.943
SC 0.1µg/mL	14.33	0.943
SC 1µg/mL	19.00	0.000
SC 5µg/mL	15.00	0.000
SC 10µg/mL	15.33	0.471
AC 0.1µg/mL	11.67	0.943
AC 1µg/mL	9.00	0.000
AC 5µg/mL	8.00	0.000
AC 10µg/mL	11.67	0.943

Supplementary Figure S3: Median Change in lifespan of worms upon treatment with SC or AC

S4

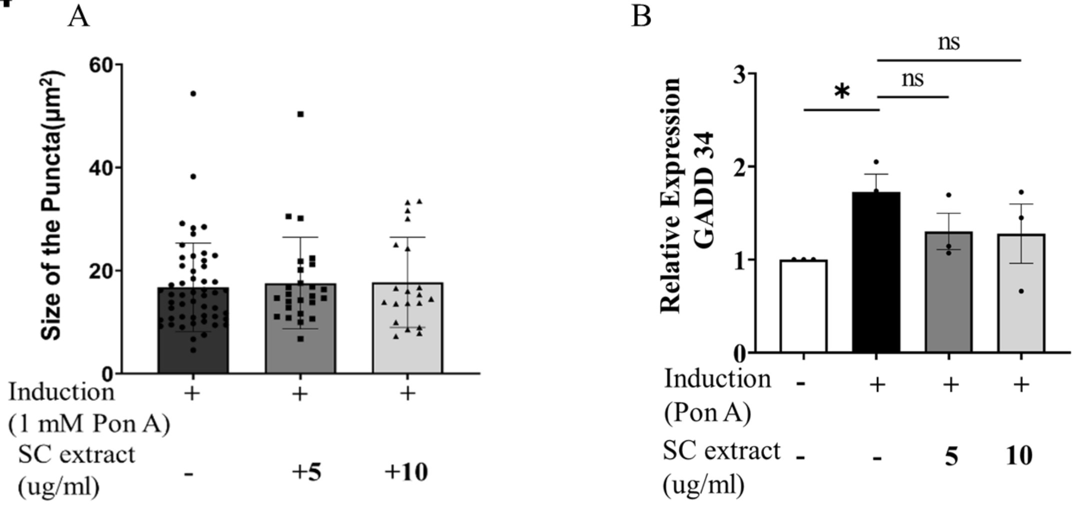

Supplementary Figure S4: A, Size of pucta upon stimulation with Ponesterone A and treatment with SC. (IC, n=52; SC 5 µg, n=25; SC10 µg, n=20, N=2) B, Relative expression GADD34 (n=3, for all the conditions) in polyQ-EGFP expressing N2a cells. *P<0.05, **p<0.01 and ns non significant. Data are mean ±SEM, n=Number of puncta taken into consideration for size calculation; N= Individual biological replicates.

S5

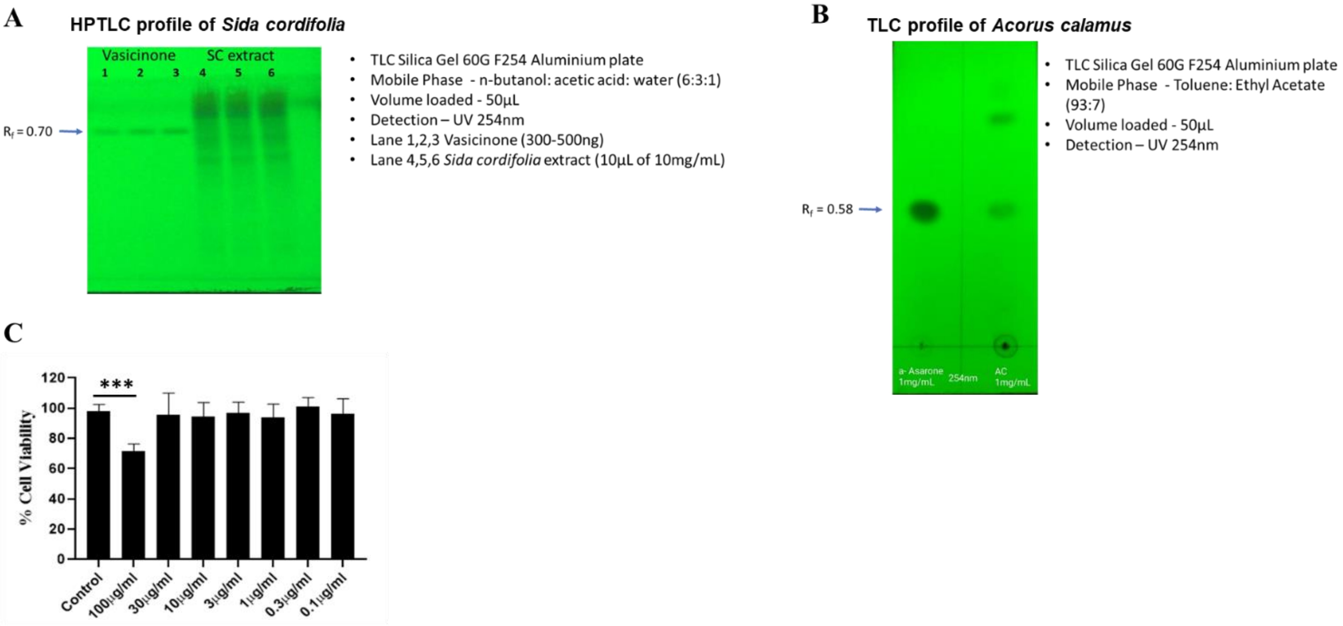

Supplementary Figure S5: HPTLC profile of SC along with marker compound Vasicinone (A). TLC profile of AC with marker compound Asarone (B). Cell viability assay of *Sida cordifolia*(SC) extract at various concentrations (C). Mean±SD. N=3. ***P ≤ 0.001(Student's T-test).

Table T1: Primers used in the study

Genes	Forward primer (5'-3')	Reverse primer (5'-3')
**XBP1 Spliced**	ACACGCTTGGGAATGGACAC	CCATGGGAAGATGTTCTGGG
**XBP1 unspliced**	GAAGAGAACCACAAACTCCAGC	GCAGAGGTGCACATAGTCTGAG
**CHOP**	CCAGCAGAGGTCACAAGCAC	CGCACTGACCACTCTGTTTC
**GADD-34**	GAGGGACGCCCACAACTTC	TTACCAGAGACAGGGGTAGGT
**BiP**	GGTACATTTGATCTGACTG	CACTTCCATAGAGTTTGCTG
**ATF4**	CCTGAACAGCGAAGTGTTGG	TGGAGAACCCATGAGGTTTCAA
**Primer for Semi quantitative PCR**		
**GFP**	ACGTAAACGGCCACAAGTCC	TGTTCTGCTGGTAGTGGTCG
**GAPDH**	GGTTGTCTCCTGCGACTTCA	TAGGGCCTCTCTTGCTCAGT

Blots used in the figures

Figure 4E

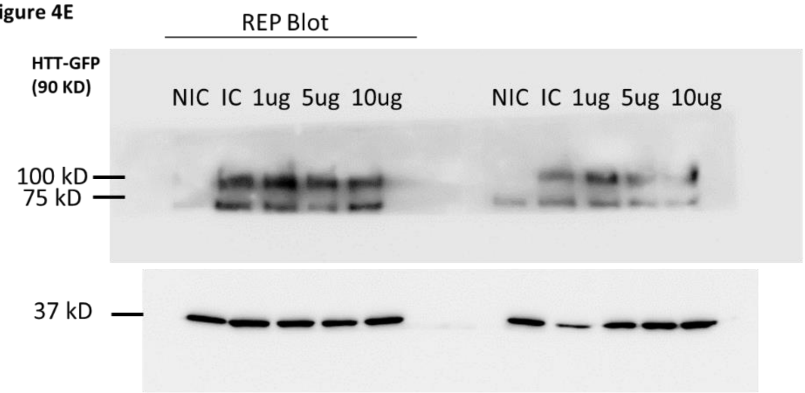

Figure 5A

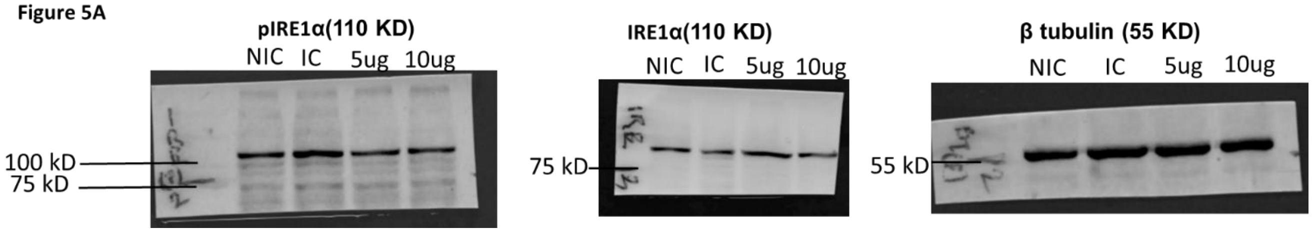

Figure 5E

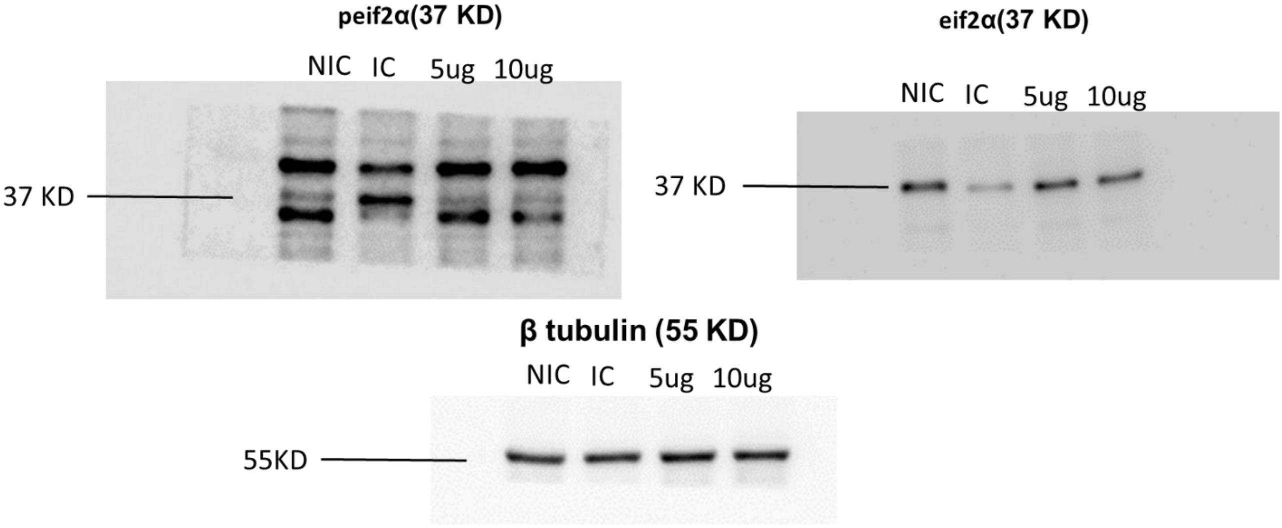

Supplement: Supplementary file 1 [file DataSheet1.pdf]
